# Initial Clinical and Cone-Beam Computed Tomography (CBCT) Outcomes of Enamel Matrix Derivative-Based Regenerative Therapy in Vertical Bone Defects

**DOI:** 10.7759/cureus.72180

**Published:** 2024-10-23

**Authors:** Tsvetalina Gerova-Vatsova

**Affiliations:** 1 Department of Periodontology and Dental Implantology, Medical University of Varna, Varna, BGR

**Keywords:** enamel matrix derivatives, initial outcomes, periodontal treatment, regenerative therapy, vertical bone defects

## Abstract

Background and objective

Enamel matrix derivatives (EMD) are biomaterials extracted from porcine dental germs. They consist of amelogenins (90%), ameloblastine, enamelin, and amelotin. New evidence has shown that all these proteins can be absorbed onto hydroxyapatite crystals and collagen fibers on the root surface of teeth and initiate regeneration of root cementum as well as stimulate periodontal cell proliferation. Currently, the most preferred method of periodontal regenerative therapy in clinical dental practice involves EMD, thanks to its facilitated surgical protocol and the many postoperative complications associated with guided tissue regeneration. There is scarce data regarding when the first and earliest signs of healing occur after the application of this method. Hence, this study focused on the evaluation and analysis of clinical outcomes and cone-beam CT (CBCT) results at six months after the application of the EMD technique. It aimed to evaluate the efficacy of regenerative therapy with EMD in vertical bone defects six months post-surgical intervention.

Material and methods

The study was conducted between August 2022 and July 2023 in the Faculty of Dental Medicine, the Medical University - Varna, using the University Medical-Dental Center as a base. The study included 12 cases characterized by bilateral, trilateral, or a combination of the mentioned vertical bone defects. The participants were aged between 31 and 57 years, of both genders, with satisfactory personal oral hygiene. They were in good general condition with no established systemic diseases and gave prior written informed consent. Between the sixth and eighth weeks after the hygiene phase was completed, the patients were re-evaluated, a new periodontal status was recorded, and a CBCT examination was ordered. Three clinical (probing pocket depth, gingival margin level, and clinical attachment level) and three radiographic parameters (A, B, and C) were evaluated in areas of vertical bone defect. Six months after the regenerative therapy using EMD, the same parameters were again recorded in all patients. Finally, after statistical processing of the results, an analysis of the data was performed regarding the early effectiveness of EMD application in regenerative therapy of vertical bone defects.

Results

The clinical results observed at the six-month mark following regenerative therapy using EMD in vertical bone defects indicated an average reduction in probing depth of 4.50 mm, an average apical migration of the gingival margin by 0.5 mm, and an average clinical attachment level of 4.00 mm. Bone filling was noted on the CBCT, corroborated by the following parameters: A - an average decrease of 1.51 mm; B - an average decrease of 0.50 mm; and C - an average decrease of 0.24 mm.

Conclusions

The results of our study confirm the remarkable effectiveness of EMD as a regenerative biomaterial. Furthermore, both clinical and CBCT studies demonstrated a very good healing process at an extremely early stage after the surgical intervention.

## Introduction

Bone tissue is endowed with the unique properties of deposition, remodeling, and turnover [1]. However, bone regenerative procedures remain an everyday challenge for periodontists, implantologists, and oral surgeons [2]. Various bone substitutes and barrier membranes have been adopted for bone grafting and reconstruction [3]. In recent years, most treatment modalities have aimed to preserve the existing bone and regenerate it in cases of deficiency [4-7]. Enamel matrix derivatives (EMD) are biomaterials extracted from porcine dental germs. Of note, they are overwhelmingly composed of amelogenins (90%), whose key role is to stimulate periodontium cell proliferation. Ameloblastine, enamelin, and amelotin are also found in significantly smaller concentrations in the composition of EMD [8]. New evidence shows that all of these low-molecular-weight proteins can be absorbed onto hydroxyapatite crystals and collagen fibers on the root surface of teeth and initiate regeneration of root cementum [9,10].

Indications for performing regenerative therapy with EMD include infraosseous bone defects (IBDs), class 2 interradicular bone defects, and single gingival recessions of class 1 and class 2 according to the Miller classification [11-13]. The European Federation of Periodontology recommends that clinicians follow the Ramfjord treatment sequence and proceed to regenerative therapy of residual IBDs only after nonsurgical treatment. For regenerative therapy, residual IBDs must be ≥3 mm [14]. Histologic findings and clinical results indicate that regeneration of periodontal structures can be achieved in infraosseous defects once various methods of regenerative therapy are applied [15]. EMD stimulates the regeneration of acellular cementum by mimicking the role of enamel proteins in cementogenesis during dental germ development [16]. Regeneration of acellular cementum is associated with the regeneration of alveolar bone and periodontal ligament [17].

Studies have reported varying results of EMD application over the years. For example, Pilloni et al. evaluated the regenerative potential of EMD (in combination with nano-hydroxyapatite) in the area of intraosseous defects. After a 24-month follow-up, an increase in the level of clinical attachment was found [18], while Ragghianti et al. reported that after 24 months of follow-up, cases were found to have significant loss of alveolar crestal bone (1.01 mm) and that the filling of the intraosseous defect was insignificant (0.04 mm) [19]. Losada et al. showed that the use of EMD with or without bone-repair material in vertical bone defects led to a significant increase in the clinical level of attachment and the level of defect filling in the 12th month after the intervention [20]. In 2004, Vandana et al. evaluated the effectiveness of EMD as a regenerative material in IBDs. The results at the ninth month post-surgery demonstrated no significant difference in the reduction of probing depth, clinical attachment level gain, and bony defect filling. This study showed that there is no additional benefit in using EMD versus surgical debridement on its own [21].

Currently, the most preferred method of periodontal regenerative therapy in clinical dental practices involves EMD, which could be attributed to its facilitated surgical protocol and the many postoperative complications linked to guided tissue regeneration (mostly barrier membrane exposition and exfoliation of bone resorption particles) [22]. Several studies in the literature have evaluated and analyzed the results of EMD application in regenerative therapy of IBDs after a long period [23-26]. However, there is scarce data regarding when the first and earliest signs of the healing process appear after the application of this method. Therefore, our study focused on evaluating and analyzing the clinical and cone-beam CT (CBCT) outcomes in the sixth month after the application of the EMD technique.

## Materials and methods

This study was conducted between August 2022 and July 2023 at Medical University Varna, specifically within the Faculty of Dental Medicine, utilizing the University Medical and Dental Center as a base. Ethical approval (№ 118/23, June 2022) was obtained from the Research Ethics Committee of the Medical University of Varna, Bulgaria. The study involved 12 cases with bipartite, tripartite, or a combination of the specified vertical bone defects. All enrolled participants were aged between 31 and 57 years, of both genders, with satisfactory personal oral hygiene. They were in good general condition with no established systemic diseases and gave their prior written informed consent. 

All patients included in the study had previously undergone the Systemic and Hygiene phases of the Ramfjord treatment sequence. Between the sth and eighth week after completion of the Hygiene Phase, their periodontal status was reassessed and a new periodontal status was recorded. At the same visit, a CBCT examination was also assigned. The method is regarded as the gold standard for volumetric evaluation of the alveolar bone [27]. Three clinical (probing depth, margo gingivalis level, and clinical attachment level) and three radiographic parameters (A, B, and C) were evaluated in the areas with the vertical bone defect. For a more precise measurement, CBCT was used and a larger number of measurements were required, as narrower and deeper intraosseous defects have a positive correlation with radiographic bone fill. Therefore, we evaluated the distance measured in mm from the enamel-cement junction to the bottom of the bone defect (parameter A), the distance measured in mm from the enamel-cement junction to the most coronally located point of the bone defect (parameter B), and the widest area of the bone defect (parameter C).

**Figure 1:**
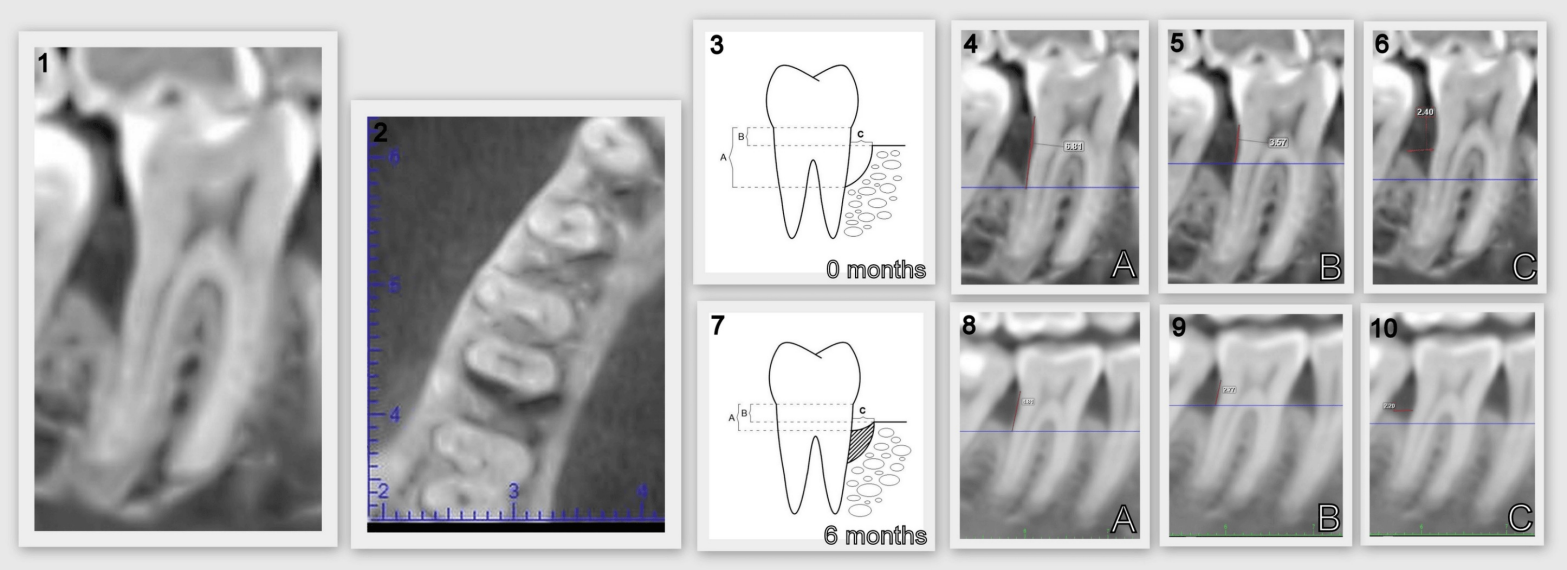
CBCT parameters (1) Coronal plane of the vertical defect on CBCT. (2) Transversal plane of the vertical defect on CBCT. (3) CBCT parameters studied before regenerative therapy (authors' own image). (4) Parameter A (0 months). (5) Parameter B (0 months). (6) Parameter C (0 months). (7) CBCT parameters studied 6 months after regenerative therapy (authors' own image). (8) Parameter A (6 months). (9) Parameter B (6 months). (10) Parameter C (6 months) CBCT: cone-beam computed tomography

We then scheduled a visit for the patients to undergo regenerative therapy using EMD. Six months after the surgical intervention, all patients had their clinical and radiographic parameters recorded again. Finally, after statistical processing of the results using analysis of variance (ANOVA), one-sample T-test, paired-samples T-Test, and one-way ANOVA, data analysis was performed regarding the early effectiveness of EMD administration in regenerative therapy of vertical bone defects. Figure 1 illustrates the CBCT parameters.

Clinical protocol of the methodology with EMD-regenerative therapy for vertical bone defects

**Figure 2:**
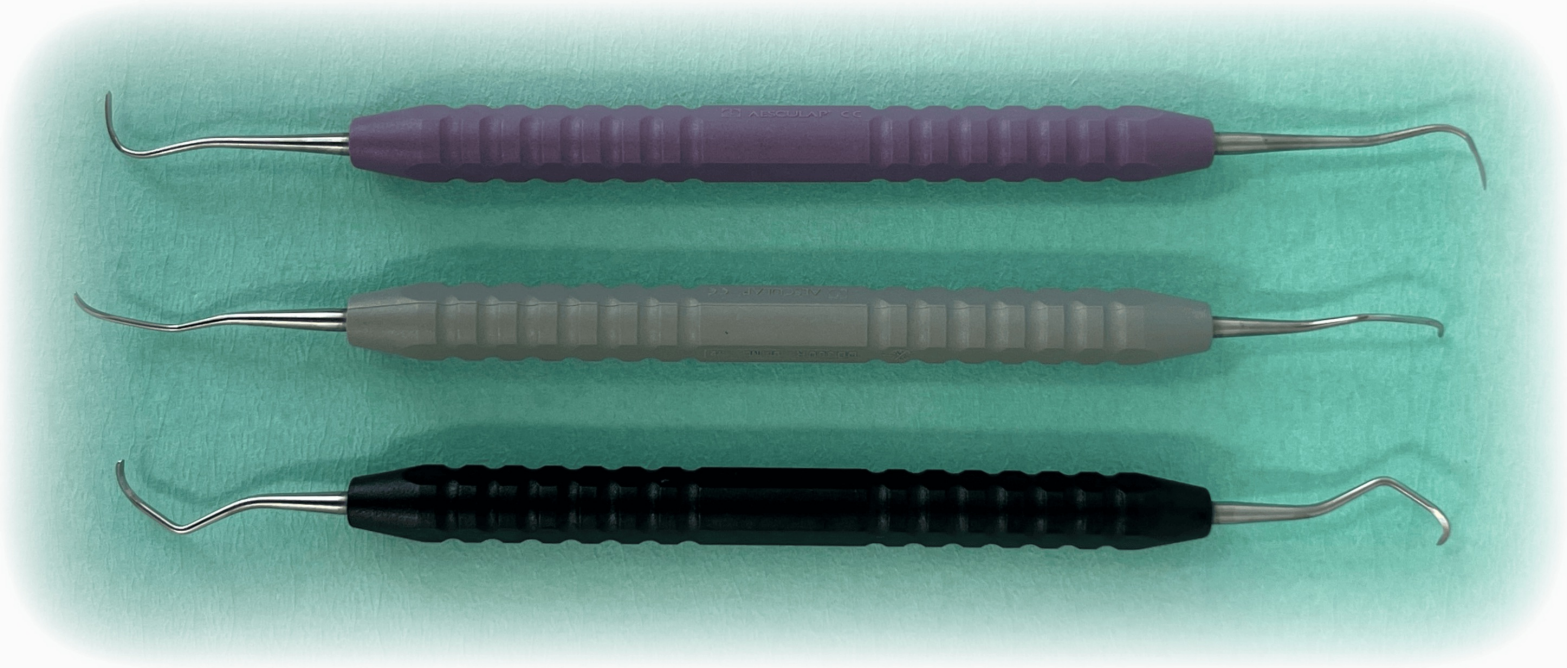
Columbia 2L/2R, Columbia 4L/4R, and Younger-Good 7/8 universal curettes

The area to be surgically treated was anesthetized by local anesthesia using Septanest (Septodont, Saint-Maur-des-Fossés, France). A scalpel was used to make an intrasulcular incision extending maximally to 1-2 teeth medial and distal to the bone defect. A mucoperiosteal flap with periosteal elevator was then reflected. Scaling and root planing were performed in the area of the bone defect using Columbia 2L/2R, Columbia 4L/4R, and Younger-Good 7/8 universal curettes (Figure 2).

**Figure 3:**
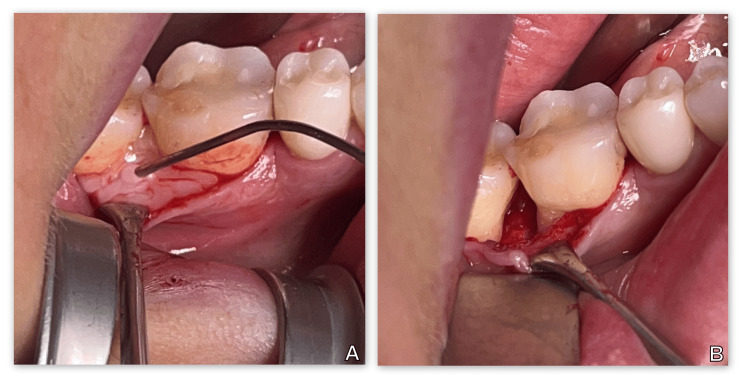
Treatment of the root surface before introduction of the EMD into the bone defect (A) Root surface treatment with 24% EDTA gel (PrefGel, Strauman). (B) After thorough washing of the EDTA-gel with saline solution EMD: enamel matrix derivative

**Figure 4:**
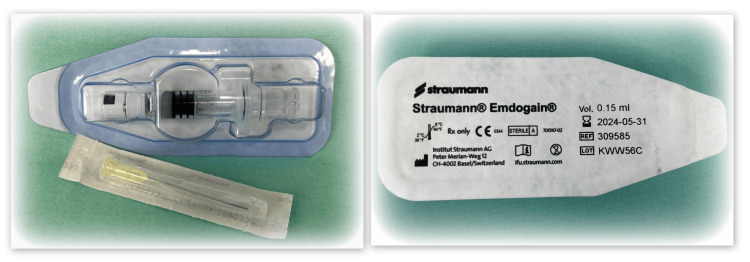
EMDs (Emdogain, Straumann) EMDs: enamel matrix derivatives

The operative field was thoroughly flushed with saline. Next, the root surface was treated with PrefGel (Strauman) (Figure 3A). After thorough washing of the EDTA gel with saline (Figure 3B), EMDs (Emdogain, Straumann) were applied to the vertical bone defect in contact with the root surface (Figure 4). This was followed by repositioning, adaptation, and suturing of the flap with 5/0 nonresorbable monofilament (Dafilon, Tuttlingen, Germany).

Postoperative care included the prescription of antibiotic medication, NSAIDs, and antibacterial mouthwash. A day and time were set for a follow-up visit and removal of suture material (10-14 days after surgery).

## Results

The parameters (clinical and radiographic) measured just before the surgical intervention of the patients (0 months) were compared with the corresponding measurements taken in the sixth month following the regenerative therapy using EMD.

Probing depth

**Figure 5:**
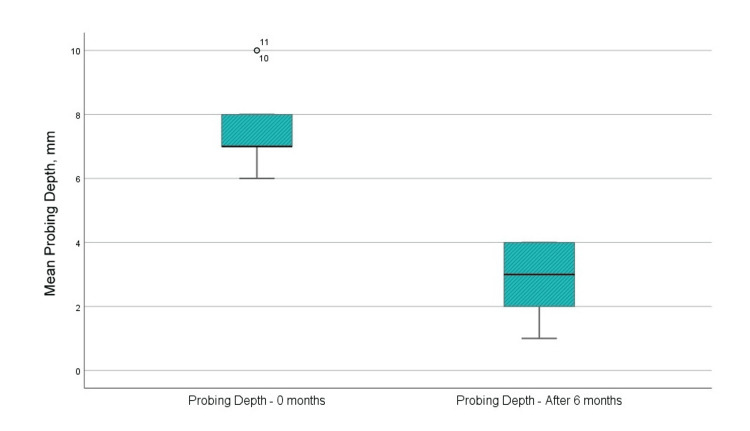
Mean values of the probing depth before the surgical intervention and at six months after the regenerative therapy

The average probing depth before surgical intervention was 7.5 mm, which diminished to 3 mm at six months post-regenerative therapy with EMD (Figure 5). The mean value of the probing depth indicator decreased by 4.5 mm, exhibiting a statistically significant difference (p<0.001).

Level of margo gingivalis

**Figure 6:**
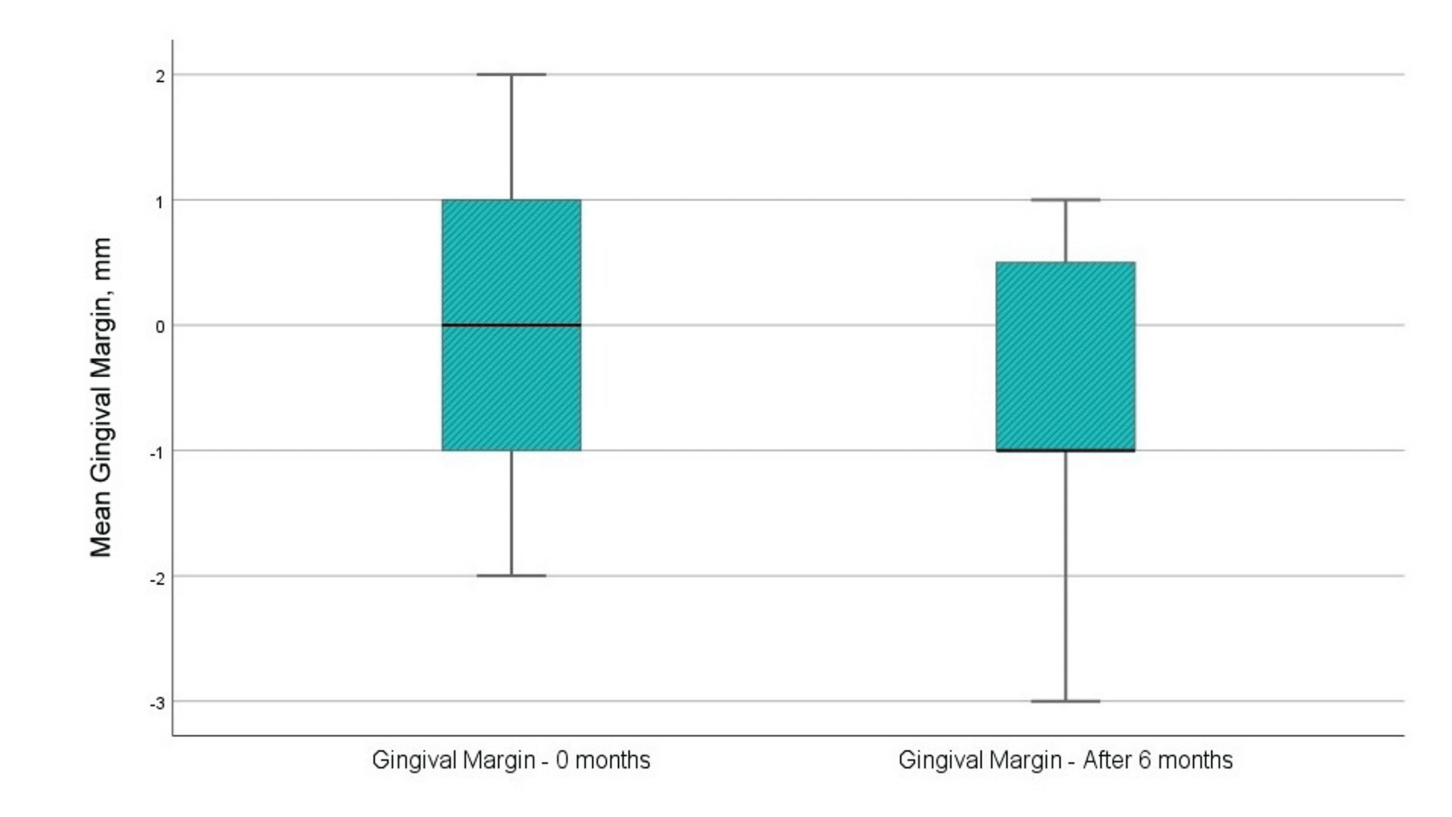
Mean values of the level of margo gingivalis before the surgical intervention and at six months after the regenerative therapy

The margo gingivalis measured -0.08 mm before surgical intervention and -0.58 mm at six months post-regenerative therapy with EMD (Figure 6). The gingival edge descended apically to the enamel-cementum junction by an average of 0.5 mm. Nonetheless, the difference was not statistically significant (p=0.041).

Clinical attachment level

**Figure 7:**
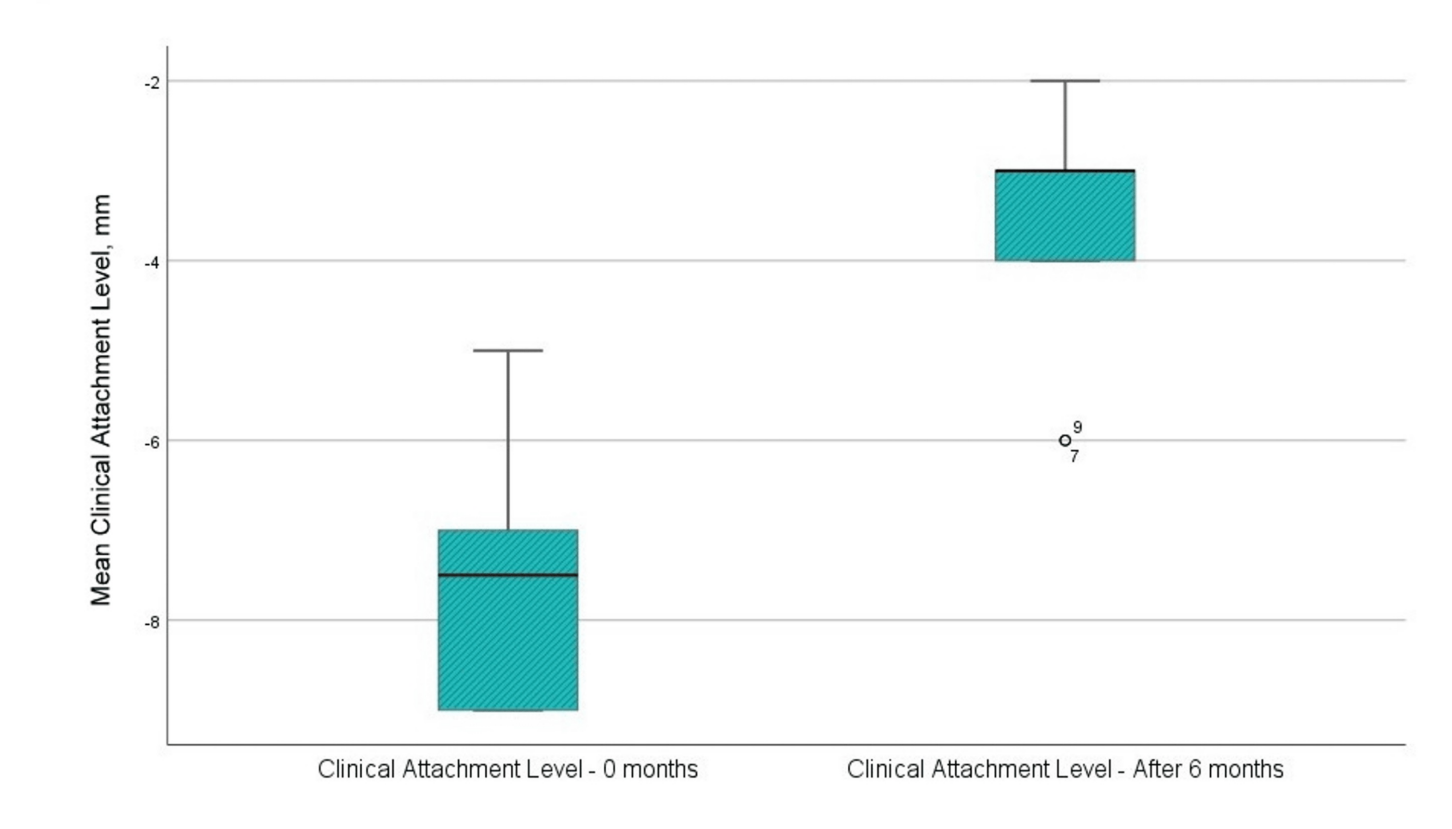
Mean values of the clinical attachment level before the surgical intervention and at six months after the regenerative therapy

The average clinical attachment level before surgical intervention was 7.58 mm, and after six months post-regenerative therapy with EMD, it measured -3.58 mm (Figure 7). The distance from the enamel-cement junction to the periodontal pocket diminished by an average of 4 mm, signifying an enhancement in clinical attachment level, and demonstrating a statistically significant difference (p<0.001).

А - Distance from enamel-cementum junction to the bottom of the bone defect (using CBCT)

**Figure 8:**
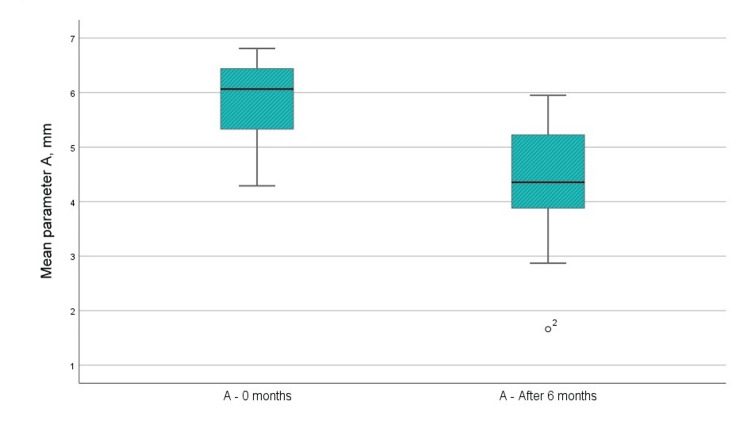
Mean values of the A parameter before the surgical intervention and at six months after the regenerative therapy

The radiographic index "A" demonstrated a notable reduction in the distance from the enamel-cement junction to the base of the bone defect after operative surgery (by 1.511 mm), signifying bone filling (Figure 8). The difference was statistically significant, with a t-value exceeding 1.796, showing a notable variation in the parameter at both 0 and 6 months post-surgery.

В - Distance from enamel-cementum junction to the apex of the bone defect (using CBCT)

**Figure 9:**
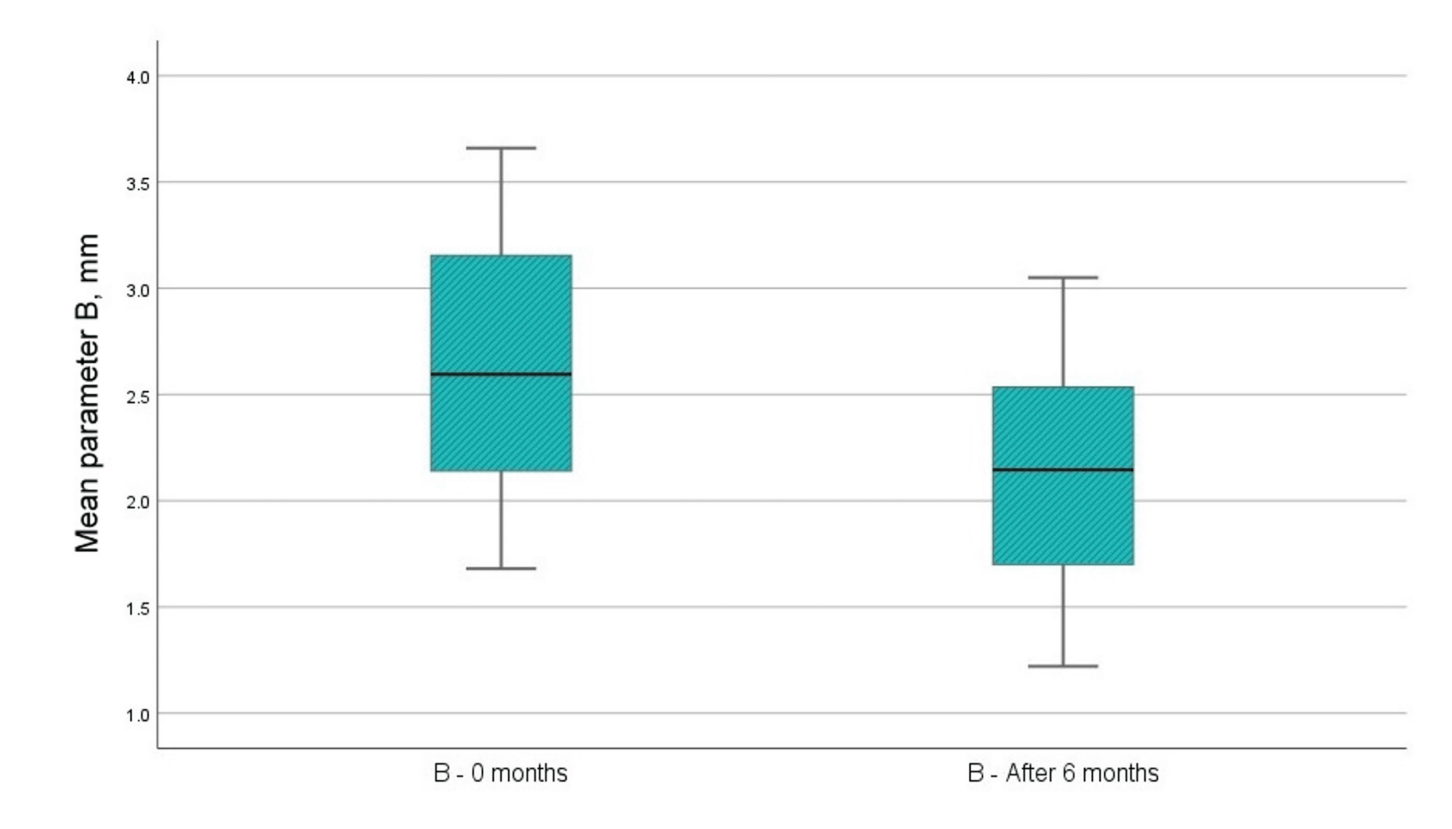
Mean values of the B parameter before the surgical intervention and at six months after the regenerative therapy

The radiographic index "B" after surgical intervention decreased by an average of 0.497 mm, indicating bone filling of the defect (Figure 9). This difference was statistically significant, with a t-value greater than 1.796, indicating that the distance from the enamel-cementum junction to the highest bone point of the defect decreased. 

С - Width of the bone defect (using CBCT)

**Figure 10:**
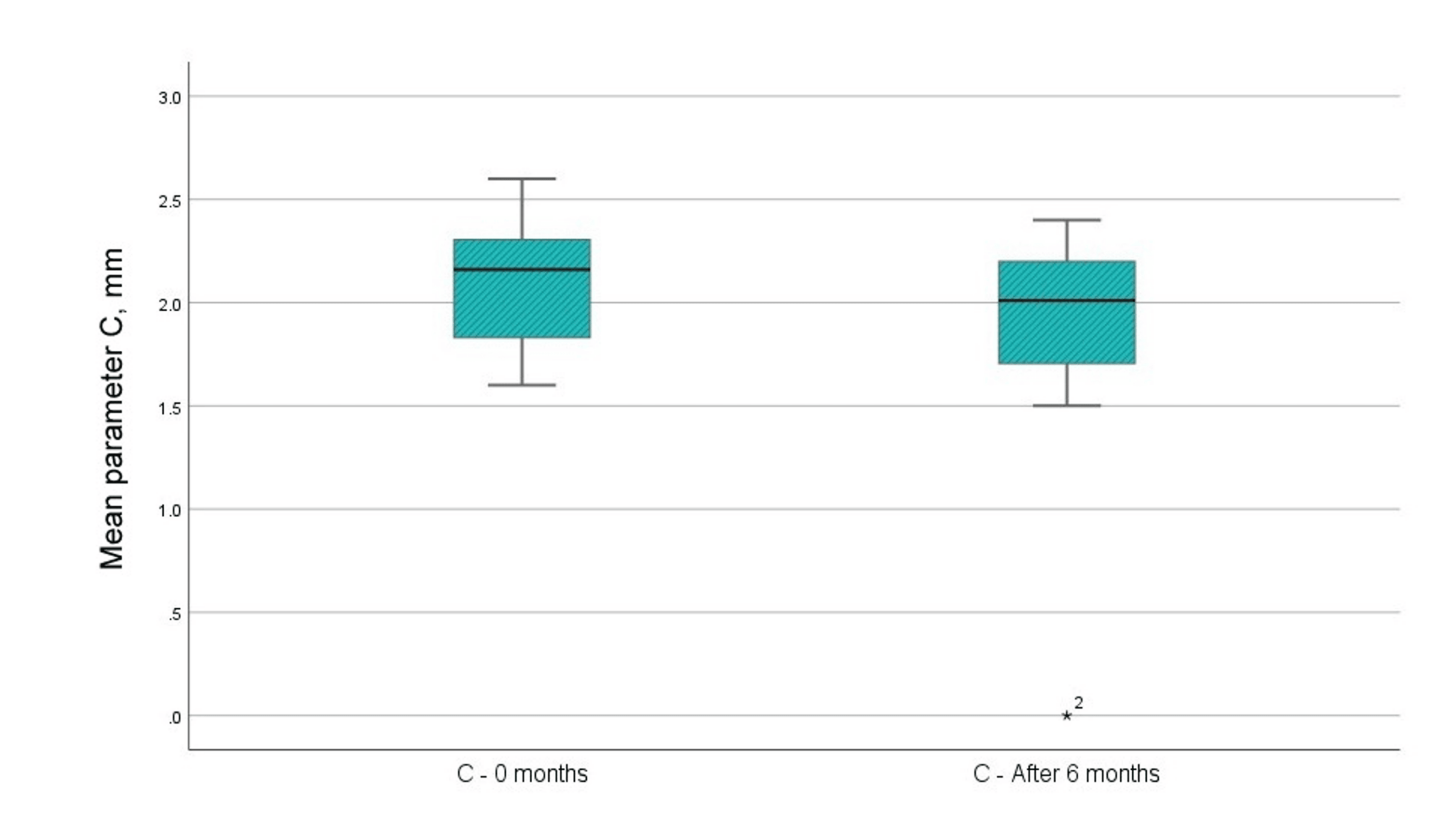
Mean values of the C parameter before the surgical intervention and at six months after the regenerative therapy

The average radiographic index "C" diminished by 0.237 mm following surgical intervention, signifying bone filling (Figure 10). Nonetheless, the disparity in the "C" parameter was not statistically significant, as the p-value was less than 1.796, with the reference value at 11 degrees of freedom.

The statistical parameters and results are presented in Table 1 and Table 2. 

**Table 1:** Paired-samples statistics with mean values and deviation

Paired-samples statistics					
		Mean	N	Std. deviation	Std. error of the mean
Probing depth	0 months	7.50	12	1.314	0.379
	6 months	3.00	12	1.044	0.302
Margo gingivalis	0 months	-0.08	12	1.165	0.336
	6 months	-0.58	12	1.240	0.358
Clinical attachment level	0 months	-7.58	12	1.311	0.379
	6 months	-3.58	12	1.311	0.379
A	0 months	5.85	12	0.822	0.237
	6 months	4.34	12	1.232	0.356
В	0 months	2.64	12	0.644	0.186
	6 months	2.14	12	0.548	0.158
С	0 months	2.07	12	0.322	0.093
	6 months	1.84	12	0.644	0.186

**Table 2:** Mean comparison and significance test

Paired-samples test									
	Paired differences							Significance	
				95% confidence interval of the difference					
	Mean	Std. deviation	Std. error of the mean	Lower	Upper	t	df	One-sided p	Two-sided p
Probing depth	4.500	1.000	0.289	3.865	5.135	15.588	11	<0.001	<0.001
Margo gingivalis	0.500	0.905	0.261	-0.075	1.075	1.915	11	0.041	0.082
Clinical attachment level	-4.000	1.595	0.461	-5.014	-2.986	-8.685	11	<0.001	<0.001
A	1.509	0.696	0.201	1.067	1.951	7.515	11	<0.001	<0.001
B	0.497	0.199	0.057	0.370	0.623	8.657	11	<0.001	<0.001
C	0.237	0.512	0.148	-0.089	0.562	1.601	11	0.069	0.138

## Discussion

EMDs are effective in stimulating the regeneration of periodontal tissue damaged after the inflammatory disease [9,10]. However, it is important to note that bone regeneration continues to increase for 36 months after the treatment of defects with EMDs [28]. Several clinical studies have evaluated and analyzed the results of EMD application in regenerative therapy of IBDs after a long period to visualize the healing processes that have occurred [23-26]. But the question remains: when is the earliest we can detect the onset of these healing processes after surgical intervention? In 2021, a study on long-term outcomes after EMD treatment showed that in IBDs, the clinical results obtained after regenerative therapy with EMD can be maintained for an average period of 10 years [25]. In 2023, Koronna et al. [26] published a study evaluating the long-term stability of outcomes after regenerative therapy with EMD alone in IBDs. They found that this regenerative approach resulted in stable outcomes over nine years.

It is important to note that narrower and deeper intraosseous defects have a positive correlation with radiographic bone fill [29]. However, due to its semi-fluid consistency, EMD has limited potential to maintain space, which in turn may lead to flap collapse in wider bone defects [30]. Therefore, the technique of guided tissue regeneration with a barrier membrane and bone repair material is recommended for wider bone defects. In this study, we worked on defects that are relatively narrower and up to 3-4 mm deep. At six months month after regenerative therapy with EMD, vertical bone defects showed a 4.50 mm decrease in probing depth, 0.5 mm apical migration of the gingival margin, and a 4.0 mm clinical attachment level. CBCT showed bone filling, as illustrated by the following parameters: A: decreased by 1.51 mm, B: by 0.50 mm, and C: by 0.24 mm.

The analysis of the "clinical attachment level" indicators at "0 months" and "six months" post-regenerative therapy with EMD revealed a mean reduction of 4.000 mm in the distance from the gingival margin to the bottom of the pocket, indicating a gain of a clinical level of attachment. Simultaneously, an analysis of the indicators "A" at the "0 months" and "six months" stages post-regenerative therapy with EMD revealed a reduction in the distance from the enamel-cement junction to the base of the bone defect, as determined by CBCT, averaging 1.509 mm. A higher percentage of soft tissue gain than regenerated bone was noted.

The analysis of our study data confirms the results obtained from several other studies in terms of validating the biologically active properties of EMD as a regenerative material. It is no coincidence that today this method is accepted as the "gold standard" in the therapeutic approach to IBDs. In addition, both clinically and on CBCT, our findings demonstrate a very good healing process at an extremely early stage after the surgical intervention (six months). 

Limitations

The main limitation of the current study that could have affected the results to some extent is the level of personal oral hygiene among the patients during these six months. In addition, studies with a larger sample size would strengthen the findings even further.

## Conclusions

Periodontal regenerative therapy procedures using EMD have a significantly easier surgical protocol compared to those of guided tissue regeneration. Moreover, postoperative complications after the application of the technique using barrier membranes and bone regenerative materials (mainly barrier membrane exposition and exfoliation of bone regenerative particles) are increasingly reported. Therefore, in the current clinical dental practice, the most preferred method of periodontal tissue restructuring involves EMD. Over the years, the efficacy of regenerative therapy using EMD has been repeatedly demonstrated. Several studies have evaluated and analyzed the results of the application of this regenerative method after a long period. Our results confirm the remarkable effectiveness of EMD as a regenerative biomaterial. Furthermore, both clinical and radiographic metrics demonstrated a very good healing process at an extremely early stage after the surgical intervention.
